# The Equine Gingiva: A Histological Evaluation

**DOI:** 10.3389/fvets.2019.00435

**Published:** 2019-12-13

**Authors:** Saskia Steinfort, Michael Röcken, Jörg Vogelsberg, Klaus Failing, Carsten Staszyk

**Affiliations:** ^1^Institute of Veterinary-Anatomy, Histology and Embryology, Faculty of Veterinary Medicine, Justus Liebig University Giessen, Giessen, Germany; ^2^Surgery, Equine Clinic, Faculty of Veterinary Medicine, Justus Liebig University Giessen, Giessen, Germany; ^3^Unit for Biomathematics and Data Processing, Faculty of Veterinary Medicine, Justus Liebig University Giessen, Giessen, Germany

**Keywords:** equine, gingiva, histology, periodontal disease, gingival tissue

## Abstract

Equine periodontal disease in horses has long been recognized as a painful disease, leading to a poor condition. The disease is widespread and attracts growing attention in equine dental medicine. The understanding of the underlying etiological and pathological mechanisms of equine periodontal disease is necessary to develop effective prophylactic and treatment options. As a first step, a thorough description of the histological features of the healthy equine gingiva is required. Specimens were taken from six horses (3 mares, 3 geldings, age: 0.5–26 years). The animals were euthanized for reasons not related to this study. Heads were dissected and gingival specimens, including parts of the adjacent teeth, alveolar bone and the periodontal ligament, were obtained from several positions of the dentition. Histological sections were evaluated via light microscopy, with special attention to the structural components of the gingiva, i.e., the gingival sulcus, the epithelium, and the components of the lamina propria (LP). Although the equine gingiva showed the same structural components as described in humans and dogs, the equine junctional epithelium was adapted to the equine dental anatomy and attached to the equine-unique peripheral cementum. Leucocytic infiltrations (LI) of the LP, sulcular epithelium (SE) and junctional epithelium (JE) were frequently seen. The amount of LI was not associated with a macroscopically visual pathology (e.g., diastema or food entrapment) in the respective position. The gingival sulcus depth had an average depth of <1 mm.

## Introduction

The equine periodontium consists of the periodontal ligament, the dental cementum, the alveolar bone, and the gingiva (1–3). Pathological alterations often start with food impaction and widening of interdental spaces, causing diastemata and periodontal pockets (4–8). A change in the composition of the oral bacterial flora causes gingivitis, which is, to a certain point, reversible (8, 9). However, if the junctional epithelium (JE) becomes destroyed, the infection spreads to the periodontal ligament, causing severe conditions of equine periodontal disease.

In human dentistry, a thorough description of the histological stages of periodontal disease is available, starting with the “initial lesion” (clinically healthy, but slightly inflamed gingival tissues), continuing with the “early lesion” (beginning gingivitis), “established lesion” (chronic gingivitis) and “advanced lesion” (periodontal disease) (10, 11). To the authors' knowledge, only one paper has provided a pathohistological description of equine periodontal disease (12). In contrast to human and canine dentistry, in which the histology of the healthy gingiva has been sufficiently described (13–17), allowing for the differentiation of pathological and non-pathological conditions, to date, no description of healthy equine gingival tissue exists.

In regard to publications in human and canine dentistry, the gingival epithelium is a parakeratinized (stratum corneum retains pyknotic nuclei) or orthokeratinized (no nuclei in the stratum corneum) stratified squamous epithelium featuring continuous regeneration due to prolonged mitosis. The gingival epithelium is connected with the underlying lamina propria (LP) by rete pegs (11, 18, 19). These are finger-shaped epithelial prolongations into the underlying connective tissue.

The sulcular epithelium (SE) is a non-keratinized stratified squamous epithelium. The cellular layers of the SE feature small gaps and therefore function as a semipermeable membrane, where gingival crevicular fluid, containing polymorphonuclear leukocytes, antibodies, enzymes, and desquamated cells, can migrate into the gingival sulcus and function as a defense mechanism against invading oral bacteria (20). The amount of gingival crevicular fluid increases with mastication and inflammation. Under pathological conditions, bacteria infiltrate the SE, and cause inflammation (18, 21–23).

The SE, bordering the gingival sulcus, merges in apical direction with the JE. This non-keratinized stratified squamous epithelium is firmly attached to the tooth and forms the bottom of the gingival sulcus. In brachydont teeth, the JE features a complex attachment apparatus, composed of hemidesmosomes, cells directly attached to the tooth (DAT cells) and the internal basal lamina (24–26), which differs from typical basement membranes, as components are synthetized by the DAT cells (25, 27) and contain a high amount of Laminin-5, which maintains cell adhesion between the JE and the tooth (28, 29). The attachment apparatus ensures a firm fixation to the enamel surface and plays a crucial role in preventing invasion of oral bacteria into the periodontal space. Noteworthy, due to the fact that the dental attachment area of the JE in horses is covered with peripheral cementum, attachment mechanisms cannot be extrapolated from brachydont species.

The interdental gingiva consists of a col-shaped part, bordered by a vestibular and palatal/lingual interdental papilla. The col is covered by a stratified non-keratinized epithelium (17, 30–32), whereas a stratified keratinized epithelium covers the interdental papillae (18). The interdental gingiva has been noted in horses (3, 21), but histological descriptions are not available. The question arises as to whether the histology of the equine interdental gingiva might be similar to other species, since equine teeth are in tight contact and the interdental space is very compressed.

Against this background, the focus of the present study is 3-fold. The first and main objective is to describe the histomorphological characteristics of the equine gingiva. Second, it is intended to report a methodical approach for further (patho-)histological investigation of different aspects of the equine gingiva. The third objective is to report histological differences and/or similarities between the equine and non-equine gingival architecture.

## Materials and Methods

**Figure 1:**
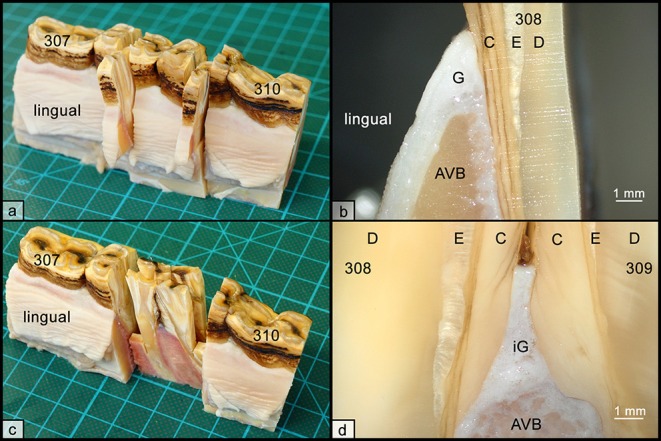
Histologic sampling collection, exemplary shown for teeth 308 and 309. **(a)** Non-interdental samples were collected by transverse sectioning of teeth 308 and 309. Sections were further divided to obtain separated specimens from the lingual and buccal aspect. **(b)** Each specimen contained the gingiva (G), parts of the alveolar bone (AVB), and dental substances, i.e., cementum (C), enamel (E), and dentin (D). **(c)** Interdental samples were collected by sagittal sections through teeth 308 and 309. **(d)** Each specimen contained the interdental gingiva (iG), attached to alveolar bone (AVB) and dental substances, i.e., cementum (C), enamel (E), and dentin (D).

Six horses (3 mares, 3 geldings, age: 0.5–24 years), euthanized for reasons not related to this study, were included (Table 1). Heads were formalin-fixed within 1 h after euthanasia. Jaws were dissected with a band saw (K440H, Kolbe Foodtec, Elchingen) and were cleaned of loose food. The presence of impacted food and diastemata were recorded. By means of a diamond saw (Proxxon Typ MBS 240/E No 27 172, Föhren, Germany), preassigned locations were cut (Table 2 and Figure 1). Sampling sites at teeth 08 and 09 (fourth premolar and first molar) were chosen because these teeth represent the youngest (08) and the oldest (09) cheek teeth within the dentition. 13 of 75 sampling sites were localized in close vicinity to diastemata or periodontal pockets. However, the sampling sites did not show any signs of gingival inflammation. Specimens were decalcified in buffered ethylene diamine tetra-acetate (EDTA, pH 8.0) for 6–8 weeks at room temperature. Afterwards, the specimens were embedded in paraffin wax, sectioned and stained (hematoxylin-eosin). Samples were assessed via light microscopy (Leica DM750 and Leica DM2500, Wetzlar, Germany, ocular magnification 10x, objectives 4x, 10x, 20x, 40x). Microscopic photographs were taken by use of a microscope camera (Leica ICC50 HD, Wetzlar, Germany). Within the figures, magnification was indicated by a scale bar.

**Table 1:** Data of horses examined.

**No**.	**Sex**	**Age**	**Breed**	**Reason for euthanasia**
1	Gelding	15 years	Welsh Cob	Laminitis
2	Gelding	26 years	Warmblood	Urolithiasis
3	Mare	8 years	Warmblood	Colic
4	Mare	9 months	Warmblood	Colic
5	Mare	26 years	Warmblood	Colic
6	Unknown	Unknown	Unknown	Unknown

**Table 2:** Preassigned locations of histologic specimen.

**No**.	**Teeth**	**Direction**	**Localization**
1	101 (I1)	Distal	Labial
2	101 (I1)	Distal	Palatal
3	101/102 (I1/I2)	Interdental	Labial
4	101/102 (I1/I2)	Interdental	Palatal
5	102 (I2)	Mesial	Labial
6	102 (I2)	Mesial	Palatal
7	108 (P4)	Distal	Buccal
8	108 (P4)	Distal	Palatal
9	108/109 (P4/M1)	Interdental	Buccal
10	108/109 (P4/M1)	Interdental	Palatal
11	109 (M1)	Mesial	Buccal
12	109 (M1)	Mesial	Palatal
13	301 (I1)	Distal	Labial
14	301 (I1)	Distal	Lingual
15	301/302 (I1/I2)	Interdental	Labial
16	301/302 (I1/I2)	Interdental	Lingual
17	302 (I2)	Mesial	Labial
18	302 (I2)	Mesial	Lingual
19	308 (P4)	Distal	Buccal
20	308 (P4)	Distal	Lingual
21	308/309 (P4/M1)	Interdental	Buccal
22	308/309 (P4/M1)	Interdental	Lingual
23	309 (M1)	Mesial	Buccal
24	309 (M1)	Mesial	Lingual

The following criterions were used to evaluate the non-interdental and the interdental gingiva (oral gingival epithelium, SE, and JE).

### Epithelium Type

Non-keratinized, orthokeratinized, and parakeratinized epithelium.

### Presence of Rete-Pegs

The specimens were observed for the presence of rete pegs (length was not considered).

### Leukocyte Infiltrations

Epithelia were evaluated for the presence of locally or diffusely distributed leukocyte infiltration (LI). The amount of LI were scored using 40x-magnification:

0: no leukocytes;
1: 1–2 leukocytes;
2: 3–10 leukocytes;
3: > 10 leukocytes.

Specific inflammatory cells (i.e., lymphocytes, plasma cells, neutrophils, macrophages, and eosinophils) were identified and the predominant cell type was determined.

### Blood Vessels

The distribution of blood vessels was recorded (locally or diffusely) and their amount was scored using 40x-magnification:

0: no blood vessels;
1: 1–2 blood vessels;
2: 3–10 blood vessels;
3: > 10 blood vessels.

### Sulcus Depth and JE Width

The sulcus depth was measured with the Leica Application Suite (LAS). The width of the JE was recorded, in which all cell layers were counted.

The following criterions were evaluated for the LP-area occlusal to a horizontal line through the apical end of the JE.

### Leucocytic Infiltration

Differentiation was made between diffusely-spread LI or local LI. Local LI were recorded, whether they were found close to the JE, the SE, or the oral gingival epithelium. The amount of LI was scored using 40x-magnification:

0: no leukocytes;
1: 1–5 leukocytes;
2: 6–50 leukocytes;
3: > 50 leukocytes.

### Blood Vessels

The distribution of blood vessels was recorded (locally or diffusely) and their amount was scored using 40x-magnification:

0: no blood vessels;
1: 1–2 blood vessels;
2: 3–10 blood vessels;
3: > 10 blood vessels.

The presence of epithelial cell rests of Malassez was recorded.

### Statistical Analysis

The statistical analyses were done by means of the statistical program packages BMDP (Dixon, W. J. (chief editor), 1993. BMDP Statistical Software Manual, Volume 1 and 2. University of California Press, Berkeley, Los Angeles, London) and StatXact (Cytel Studio StatXact Vers. 9.0.0 (2010), Statistical Software for Exact Non-parametric Inference, User Manual. CYTEL Inc., Cambridge, MA 02139, U.S.A.). The description of the data was done by forming two-way frequency tables including absolute and relative frequencies (program BMDP4F). For the dichotomous target variables the groups (macroscopically diseased or not) were statistically compared by means of the Fisher exact test (program BMDP4F) and for ordinal scaled variables using the exact Wilcoxon rank-sum test due to many ties in the data (program StatXact).

In all cases, a significance level of α = 0.05 was used.

## Results

The histomorphological characteristics of the equine gingiva (Table 3) did not differ between tooth type (incisors, premolars and molars) nor between upper and lower jaw and nor between localizations along the dental circumference (vestibular, lingual/palatal, mesial, distal).

**Table 3:** Characteristics of the histology of the equine gingiva.

**NON-INTERDENTAL GINGIVA**	
Oral epithelium	
Parakeratinized stratified squamous epithelium	84.1%
Orthokeratinized stratified squamous epithelium	15.9%
Presence of rete-pegs	100%
Leukocyte infiltration	0%
Sulcular epithelium	
Non-keratinized stratified squamous epithelium	100%
Presence of rete-pegs:	
- Full length of the SE	67.6%
- In the occlusal half	16.2%
No leukocyte infiltration:	38.4%
Category 1 leukocyte infiltration (1–2 leukocytes):Category 2 leukocyte infiltration (3–10 leukocytes):	13.7% 47.9%
Sulcus depth	
- Range:	0.1 to 2.7 mm
- Mean:	0.8 mm
- Standard deviation:	0.5 mm
**Junctional epithelium**	
Stratified squamous epithelium	100%
Presence of rete-pegs:	0%
No leukocyte infiltration:	66.7%
Category 1 leukocyte infiltration (1–2 leukocytes):	6.1%
Category 2 leukocyte infiltration (3–10 leukocytes):	15.1%
Category 3 leucocyte infiltration (>10 leukocytes):	12.1%
**Lamina propria**	
Local leukocyte infiltration	39.1%
Diffuse leukocyte infiltration	60.9%
Diffuse blood vessel infiltration	
- Category 1 (1–2 blood vessels):	1.3%
- Category 2 (3–10 blood vessels):	84,0%
- Category 3 (>10 blood vessels):	14.7%
**INTERDENTAL GINGIVA**	
**Epithelium**	
Non-keratinized stratified squamous epithelium:	15.8%
Non-keratinized stratified squamous epithelium but sporadic corneocytes:	68.4%
Keratinized stratified squamous epithelium:	15.8%
Rete-pegs at occlusal tips only	100%
No leukocyte infiltration:	95.6%
Category 2 leukocyte infiltration locally (3–10 leukocytes):	4.3%
**Lamina propria**	
Leukocyte infiltration close to the epithelium and additionally diffuse infiltration	48.0%
Diffuse leukocyte infiltration only	28.0%
Local leukocyte infiltration only	24.0%
Blood vessels category 2 (3–10 blood vessels)	100%

### Oral Gingival Epithelium

**Figure 2:**
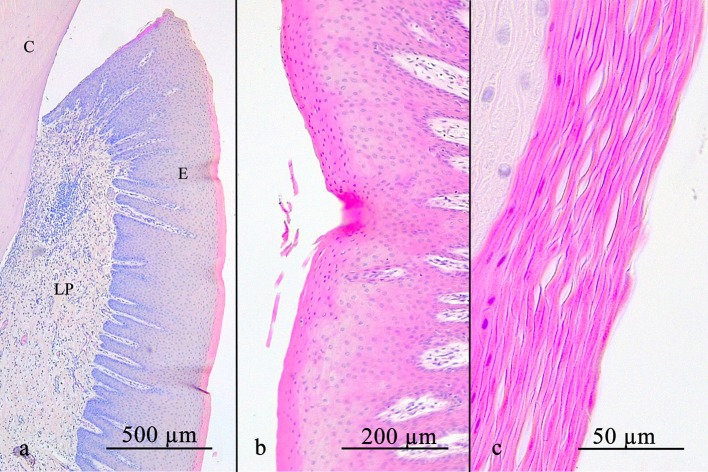
**(a)** shows an overview of the stratified squamous oral gingival epithelium with its rete pegs. Notice the decreasing width of the stratum corneum occlusally. The oral gingival epithelium can either be parakeratinized **(b)** or orthokeratinized **(c)**. C, cementum; E, gingival epithelium; LP, lamina propria.

A parakeratinized stratified squamous epithelium was present in 58 of 69 positions (84.1%), in the remaining 15.9%, a partially orthokeratinized stratified squamous epithelium was found. The keratinization and number of cell-layers decreased in the occlusal direction. Rete pegs were present in all specimens. No LI and blood vessels were noted (Figure 2).

### Sulcular Epithelium

In all cases, the SE featured a non-keratinized stratified squamous epithelium (72/72). Rete pegs were found in 67.6% (46/68) at the full length of the SE. In 16.2% (11/68), rete pegs were only recorded in the occlusal half. No rete pegs were observed in 16.2% (11/68). In 10 of 73 positions (13.7%), a small number of LI (category 1) were determined locally. In 47.9% (35/73), a moderate number of LI (category 2) were recorded diffusely. LI were predominantly neutrophils. No LI were recorded in 38.4% (28/73). No blood vessels were recorded.

### Gingival Sulcus

The sulcus depth ranged from 0.1 to 2.7 mm, average sulcus depth 0.8 mm, standard deviation 0.5 mm.

**Figure 3:**
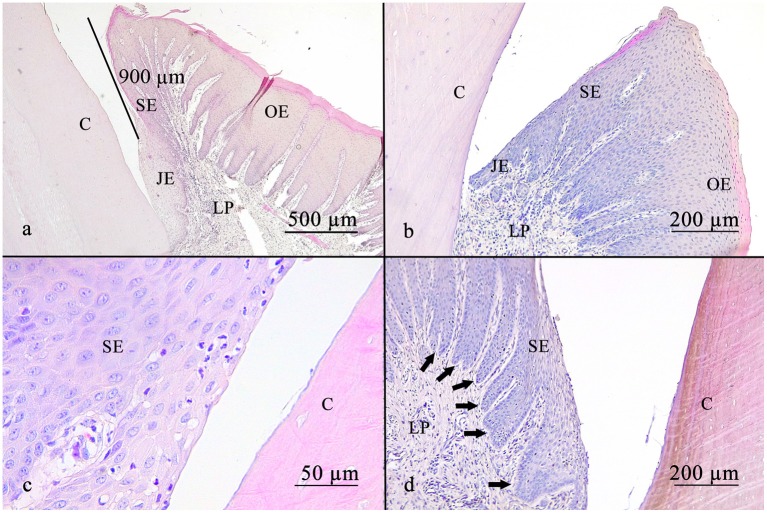
The gingival sulcus was measured from the gingival margin to the suspected bottom of the gingival sulcus **(a)**. The average depth was <1 mm. The sulcular epithelium is a non-keratinized epithelium **(b)**. Leucocytic infiltrates are able to get through the sulcular epithelium, as shown in **(c)**. Rete pegs are visible **(d)** (arrows). C, cementum; JE, junctional epithelium; SE, sulcular epithelium; OE, oral epithelium; LP, lamina propria.

In 13.4% (9/67), the sulcus measured <0.5 mm. In 55.3% (37/67), the sulcus measured between 0.5 and 1 mm. In 31.3% (21/67), the sulcus measured more than 1 mm (Figure 3).

### Junctional Epithelium

**Figure 4:**
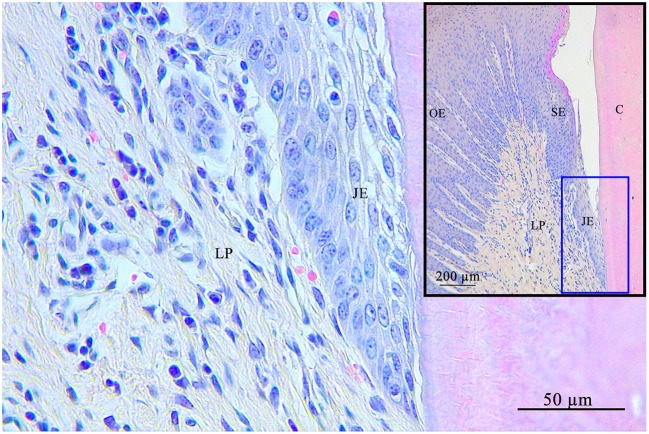
Junctional epithelium (blue square) at the bottom of the gingival sulcus. The junctional epithelium is the connection of the gingiva with the tooth. C, cementum; JE, junctional epithelium; SE, sulcular epithelium; OE, oral epithelium; LP, lamina propria.

The JE always featured a stratified squamous epithelium lacking rete pegs and blood vessels. In 6.1% (4/66) LIs were present and assigned to category 1, in 15.1% (10/66) to category 2, and in 12.1% (8/66) to category 3. No LI were recorded in 66.7% (44 /66). The number of cell layers decreased in apical direction (Figure 4).

### Lamina Propria

**Figure 5:**
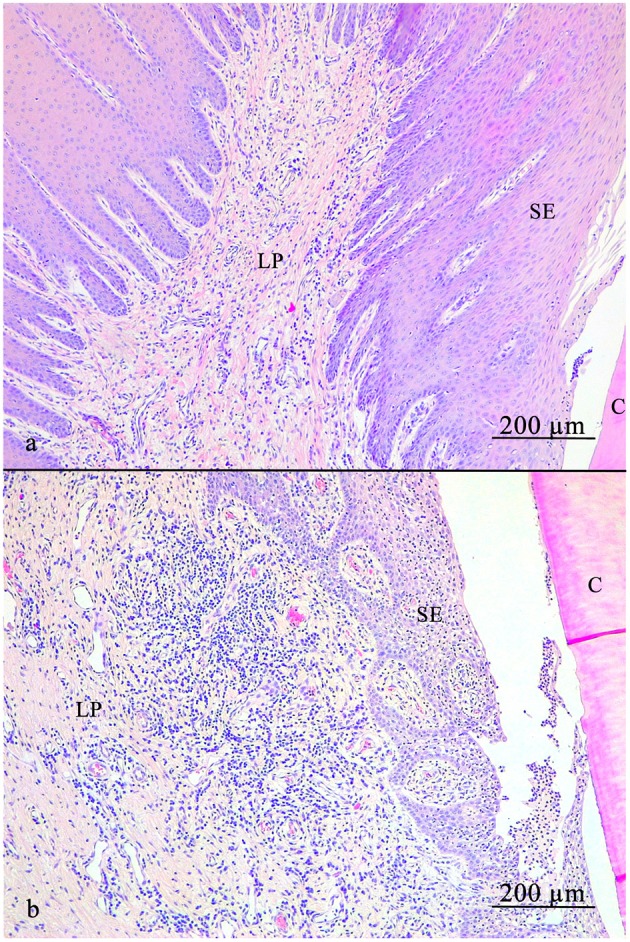
Lamina propria of the gingiva. **(a)** Note the diffusely spread blood vessels. **(b)** Local accumulation of leucocytic infiltrates near the sulcular epithelium (SE). C, cementum; SE, sulcular epithelium; LP, lamina propria.

The LP (Figure 5) always contained LI, dominated by lymphocytes with lesser contents of plasma cells, macrophages and neutrophils. No eosinophils were observed.

In 39.1% (43/110) LI were found locally, close to the epithelia and most of them (72.1%) were assigned to category 3 (31/43). Additional diffuse LI were assessed in 60.9% (67/110). Of those, 22.4% (15/67) were assigned to category 1, 65.7% (44/67) to category 2 and 11.9% (8/67) to category 3. Samples taken from sites next to a diastema or periodontal pocket did not show significant differences in terms of LI compared to other samples (*p* > 0.05). Diffusely-spread blood vessels were observed in every sample. In 84.0% (63/75) a blood vessel amount according to category 2 was recorded, 1.3% (1/75) category 1, and 14.7% (11/75) category 3. Epithelial cell rests of Malassez were present in 20.3% (15/74).

### Interdental Gingiva

In 68.4% (13/19), the interdental epithelium was non-keratinized, but sporadic corneocytes were found. In 15.8% (3/19), a keratinized stratified squamous epithelium was recorded and in 15.8% (3/19), the epithelium showed a non-keratinized stratified squamous epithelium. The epithelium consisted of more than 10 cell-layers in the region of the occlusal tip of the interdental gingiva. In the mesial and distal direction, approaching the dental surfaces, the number of epithelial cells decreased and adjacent to the dental surface a line of single cells attached the tooth, resembled a JE.

Rete pegs were seen in all samples at the occlusal tip of the interdental gingiva but were absent next to the dental surfaces. LI (category 2) were present in 4.3% (1/23) close to the LP. No epithelial blood vessels were present.

**Figure 6:**
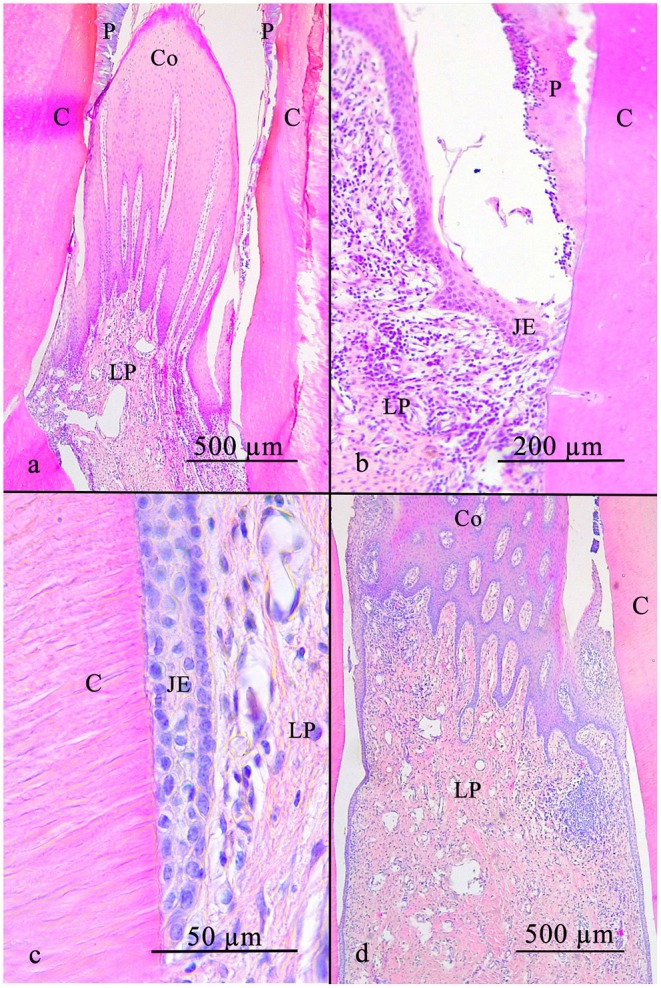
The equine interdental gingiva is cone shaped and has a non-keratinized stratified squamous epithelium with rete pegs **(a)**. In direction of the tooth the epithelium flattens **(b)** and finally connects to the tooth **(c)**. The lamina propria includes leucocytic infiltrates in a low number spread all over the connective tissue and in high numbers locally near the epithelium **(d)**. C, cementum; JE, junctional epithelium; LP, lamina propria; P, plaque; Co, Col.

The LP always contained blood vessels (amount according to category 2) and was consistently infiltrated with leukocytes. LI (category 3) close to the epithelium and additionally diffuse infiltration (category 2) were present in 48.0% (12/25). In 28.0% (7/25) only diffuse LI were recorded, and in 24 % (6/25) exclusively local infiltration was seen. No epithelial cell rests of Malassez were present (Figure 6).

## Discussion

Although diseases of the gingiva and deeper periodontal structures are recognized as a major problem in equine dentistry, the pathogenesis remains widely not understood. Recently, Cox et al. (12) suggested an overlap of normal histological and pathohistological features of the equine gingiva. However, hitherto, no investigations have been performed to describe the histomorphological features of the healthy equine gingiva. Instead, data obtained from non-equine brachydont species had been adapted as a basis for diagnosis, therapy and histopathological investigations of equine gingival diseases (1, 3, 12, 21, 22). The present study aims to elucidate equine specific characteristics of the gingiva in order to provide a basis for further histopathological investigations (12).

The findings of the equine oral gingival epithelium are predominantly consistent with descriptions of the human and canine gingiva. The oral gingival epithelium of humans and dogs is a parakeratinized stratified squamous epithelium (33–37), but can also be orthokeratinized (37–39). The degree of keratinization is not only genetically determined, but also a result of mechanical irritation (39). Interestingly, the oral gingival epithelium loses its keratinization and change its histomorphological features toward a sulcular epithelium when experimentally moved into the gingival sulcus (38, 39). Rete pegs are present in human and canine oral gingival epithelium, just as in the horse (23, 36, 37, 40). In humans and dogs, long rete pegs indicate a tight and healthy connection between the epithelium and the underlying connective tissue, manifested as macroscopically visible impressions on the gingival surface, so called “stipplings” (37, 41–43). “Stippling” was not assessed in the present study. This might be due to the fact that only cadaveric heads were investigated. Further studies are needed to clarify whether “stippling” is also a constant feature in the living horse and whether, “stippling” might be a useful criterion to identify early pathological changes, as suggested for humans and dogs (37, 44, 45). No blood vessels or leucocytic infiltrations were found in the equine oral gingival epithelium; this is consistent with reports in non-equine species.

The equine SE featured a non-keratinized stratified squamous epithelium. However, in humans, a large variety of the SE is described. Usually, a non-keratinized stratified squamous epithelium is present (18, 36, 46), but tendencies of keratinization were found (47). Investigations by Caffesse et al. (38, 39, 48) showed that the SE is able to keratinize when exposed to mechanical stimulation. The equine SE showed rete pegs in the majority of cases. In the human SE, rete pegs have been described as a constant feature (18, 39). Nonetheless, other investigations deny the presence of rete pegs in the SE (46). In the present study, neutrophils were found in the healthy SE in 61.6% of the samples. This is in line with findings obtained in the horse (12), and in humans and dogs, in which the presence of the neutrophils in the healthy SE was described as a common feature (49–53). Interestingly Cox et al. (12) found a significant association between the appearance of intraepithelial neutrophils and equine periodontal disease.

In the present study, the JE featured a stratified squamous epithelium without rete pegs. This is consistent with data from brachydont species (14, 17, 54). Hull et al. (55) and Nanci and Bosshardt (20) indicated that, with progressing inflammation, the JE elongates and proliferates forming rete pegs. The structure of the JE, which provides firm attachment to the enamel dental surface, is extensively described for the brachydont teeth. The main features are the internal basal lamina, DAT cells and specialized hemidesmosomes (25, 35). Due to the fact that the equine hypsodont tooth shows marked differences compared to the brachydont tooth (cemental surface instead of an enamel surface, continuous eruption rather than temporary eruption), the gingival attachment apparatus is hypothesized to show equine-specific adaptions and is required for continuous remodeling. In this respect, the shown capacities of the human JE to regenerate and for *de novo* formation might serve as a basis for further investigations in the horse (56, 57).

LI (predominantly neutrophils) were regularly seen in the JE of equine samples. As known from human and canine dentistry, neutrophils infiltrate the JE, which has comparatively less desmosomes than other oral epithelia (58, 59). Attström and Egelberg (60), Klinge et al. (61), Schroeder (62), Preshaw (11) i.a. described the incidence of neutrophils, lymphocytes, monocytes or rather macrophages and IgG-, IgM-, IgA antibodies as part of the immune response against oral bacteria. The leukocytes do not stay within the JE, but transmigrate into the gingival sulcus, forming the crevicular fluid. Interestingly, the crevicular fluid flow and content is used to stage PD in humans and dogs (14, 63). Further studies will show whether such a diagnostic approach is also feasible for the early diagnosis of equine periodontal disease.

The LP in the present study showed a large number of inflammatory infiltrates, dominated by high contents of lymphocytes and minor contents of other leukcoytes. This is in accordance with the only available publication in equine dentistry, where mainly lymphocytes, plasma cells, and occasionally eosinophils, were described (12). In man, neutrophils, lymphocytes, macrophages and plasma cells were noted (11, 17, 61, 64). In none of the investigated epithelia (oral gingival epithelium, SE, and JE) blood vessels were detected. Although the presence of intraepithelial blood vessels appears to be very unlikely, such a particular feature is well known from the stria vascularis (65). This specialized epithelium within the inner ear plays a major role in the production of the endolymph (66). Since the SE and especially the JE are also involved in the production of an extracellular fluid, i.e., the crevicular fluid, there was good reason to proof the existence of intraepithelial blood vessels in the equine gingiva.

In the present study, LIs were almost always seen close to the SE and JE. Additionally, milder, diffuse infiltrations were found all over the LP. This pattern has regularly been found in non-equine species, as described by Nanci and Bosshardt (20) and Freedman et al. (49). Cox et al. (12) also recognized this pattern in equine samples, describing two areas: the superficial LP, which contains lymphocytes, plasma cells and eosinophilic neutrocytes, and the deeper LP, which is mainly infiltrated by lymphocytes and plasma cells. This infiltration is usually milder than in the superficial LP. However, no correlation between LI and equine periodontal disease was detected by Cox et al. (12).

In the present study, blood vessels were found throughout the LP, with no local accumulations. For other species (dogs, rats, monkeys, and humans), two blood vascular networks are described. Capillary loops close to the oral epithelium, and a network close to the JE, consisting of anastomosing blood vessels, which feature characteristics of high endothelial venules, allowing diapedesis (17, 67–69). Future studies using techniques to display the ultrastructural components of blood vessels are required to search for similar vascular structures in the equine gingiva.

The histomorphological features of the interdental gingiva obtained in our study were similar to those described for man and dog. The epithelium visible in interdental samples is described to be JE and SE, which fuses at the col (17, 31, 32). Kohl and Zander (30) described a non-keratinized squamous stratified epithelium at the col, with increasing cell layers toward the middle (31). In contrast to the findings in our study, where more than 10 cell layers were counted, <10 cell layers are described in man (30, 31). A stratified keratinizing epithelium, as occasionally found in our study, is not described in brachydont species. A possible explanation might be a foregone irritation by entrapped forage, which is able to cause keratinization, as shown by Caffesse et al. (38, 39, 48). This finding indicates the important role of gingival defense mechanisms, especially in the interdental region, where most equine periodontal disease are initiated (8, 70).

## Conclusions

The presented methodical approach has been proven to provide suitable histological specimens for the description of the healthy equine gingiva. The histomorphological characteristics of the equine gingiva appear to be quite similar to humans and dogs, despite the differences of brachydont and hypsodont teeth and the very different diets. The obtained results might serve as a basis to establish a histological grading system for the equine periodontal status. Nonetheless, further studies are necessary to advance prophylactic and therapeutic treatments in equine periodontology.

## Data Availability Statement

The datasets generated for this study are available on request to the corresponding author.

## Ethics Statement

Ethical review and approval was not required for the animal study because Six horses (3 mares, 3 geldings, age: 0.5–24 years), euthanized for reasons not related to this study, were included. Written informed consent was obtained from the owners for the participation of their animals in this study.

## Author Contributions

CS and MR contributed conception and design of the study. JV and SS organized the database. KF performed the statistical analysis. SS wrote the first draft of the manuscript. All authors contributed to manuscript revision, read, and approved the submitted version.

### Conflict of Interest

The authors declare that the research was conducted in the absence of any commercial or financial relationships that could be construed as a potential conflict of interest.
